# Consumers’ satisfaction factors mining and sentiment analysis of B2C online pharmacy reviews

**DOI:** 10.1186/s12911-020-01214-x

**Published:** 2020-08-17

**Authors:** Jingfang Liu, Yingyi Zhou, Xiaoyan Jiang, Wei Zhang

**Affiliations:** 1 School of Management, Shanghai University, 99 Shangda Road, Shanghai, 200444 China; 2 School of Economics & Management, Tongji University, Shanghai, China

**Keywords:** B2C, Online pharmacy, Online review, Topic mining, Sentiment analysis

## Abstract

**Background:**

In recent years, online pharmacies have been accepted by increasingly more consumers, and the prospects for online pharmacies are optimistic. This article explores the consumers’ satisfaction factors addressed in Business to Customer (B2C) online pharmacy reviews and analyzes the sentiments expressed in the reviews. The goal of this work is to help B2C online pharmacy enterprises identify consumers’ concerns, continuously improve the health services level.

**Methods:**

This article was based on the Latent Dirichlet Allocation (LDA) topic model. From a third-party platform-based B2C online pharmacy and a proprietary B2C online pharmacy (JD Pharmacy and J1.COM, respectively), 136,630 pieces of over-the-counter (OTC) drug review data posted from January 1, 2015 to December 31, 2018 were selected as samples and used to explore the satisfaction factors of B2C online pharmacy consumers regarding the entire drug purchasing process. Then, the sentiments expressed in the drug reviews were analyzed with SnowNLP.

**Result:**

Categorization of the 12 factors identified by LDA showed that 5 factors were related to logistics; these 5 factors, which also included the most drug reviews, made up 38.5% of the reviews. The number of factors related to drug prices was second, with 3 factors, and reviews of drug prices made up 25.5% of the reviews. Customer service and drug effects each had two related factors, and a smaller percentage of these reviews (13.95%) were related to drug effects. Consumers still maintain positive opinions of JD Pharmacy and J1.COM. However, some opinions on logistics and drug prices are expressed.

**Conclusion:**

The most important task for online pharmacies is to improve logistics. It is better to develop self-built logistics. Both types of B2C online pharmacies can improve consumer viscosity by implementing marketing strategies. With regard to customer service, focusing on improving employees’ service attitudes is necessary.

## Introduction

### Background

The Internet has completely changed the way in which we live and communicate, and it has also changed the methods and strategies people use to procure necessary items [1]. As Internet access increases, the need to search for health information also increases all over the world [2–4]. A recent article found that nearly half of Americans first consulted the Internet for information about health or medical problems [5]. The use of mobile devices with portability, mobility, personalization and ubiquity has further amplified this trend [6, 7].

Consumers not only retrieve health information from the Internet but also obtain a variety of health services or products [8, 9]. With the continuous expansion of the digital health industry, the pharmaceutical e-commerce has developed rapidly [10]. B2C online pharmacies may be online branches of offline pharmacies or third-party B2C platforms which provide virtual transaction platform services for the consumers and the drug sellers in a neutral identity [11–13]. The excellent consumer experience and the convenience of transactions during online shopping have contributed to the growing market share of online pharmacies [14, 15]. Online pharmacies have also encountered many problems in their operations [16]. For example, because of the unwillingness of many illegal websites to disclose their actual locations, it is impossible to establish an effective regulatory framework for Internet pharmacy logistics operations [17, 18]. According to The World Health Organization (WHO), 10% of drugs sold globally through online suppliers may be counterfeit [17, 19, 20].

Early reports show that there were very few actual cases in which prescription drugs were purchased online [21]. However, recent reports indicate that the number of people who use Internet pharmacies to purchase drugs and other online health products is increasing [22]. Although the scale of the online drug sales market in China showed a significant increase between 2012 and 2018, it still accounts for 9.1% of the total retail drug market share in 2018. Compared with the drug retail market in the US, of which e-commerce represents 33.3%, China’s pharmaceutical e-commerce still has much room for growth [23].

### Related work

Pharmaceutical e-commerce is the product of e-commerce development. B2C pharmaceutical e-commerce is a kind of business activities related to pharmacies relying on network technology between online pharmacies and consumers [24].Consumers’ reviews on the e-commerce website are evaluations of the products or services obtained by consumers who purchase products or services [25], and consumer reviews provide information for other consumers to select and purchase products [26]. By reading reviews, a consumer can reduce his or her uncertainty about a product or service [27]; at the same time, online pharmacy’s reviews can attract more potential consumers to the site, increase consumer access time on the site, and increase consumer stickiness to the site [28]. Vermeulum and other scholars pointed out that positive consumer reviews will have a positive impact on potential customers of a hotel [29]. FISKE pointed out that the emergence of negative evaluations in the social environment will attract consumers’ attention and have a negative impact on product sales [30]. Duan conducted a study on film reviews, pointing out that consumer reviews have important persuasiveness and propaganda effects on movie box offices and should be considered internal indicators [26]. This shows that consumer reviews have significant business value. Guo identified the key dimensions of customer service that hotel customers care about by mining the reviews on hotel websites. These dimensions have important reference value for hotel customer service improvement [31].

Current research on online pharmacy reviews includes the use of Analytic Hierarchy Process (AHP) to conduct comprehensive quality assessments of online pharmacies, but the results of AHP are greatly influenced by subjective judgments [32]. The chameleon clustering algorithm was used to cluster hot reviews, but the complexity of the algorithm made the calculation too time-consuming to complete [33]. The corresponding analysis method has been used to study the differences among pharmaceutical e-commerce websites, but the number of samples collected, especially the number of negative reviews, was very small, and this number may have affected the results of the analysis [34].

## Methods

### Research framework

**Fig. 1:**
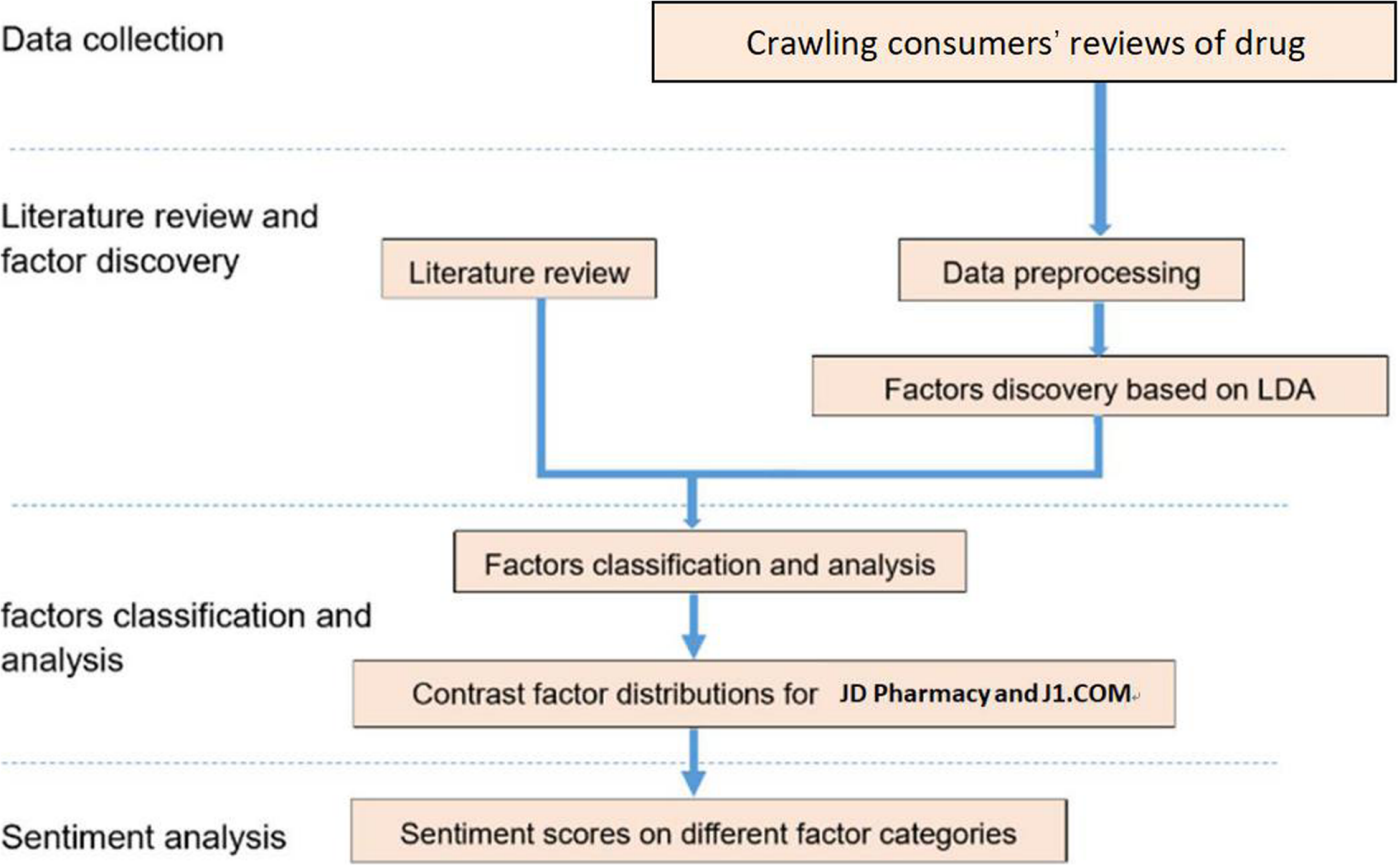
Methodological framework

The purpose of this article was to mine and analyze reviews of the entire transaction process submitted by consumers of two B2C online pharmacies. Chinese B2C online pharmacies are mainly divided into third-party platform-based B2C online pharmacies and proprietary B2C online pharmacies. Third-party platform-based B2C online pharmacies mainly refer to the third-party B2C platforms, which provide virtual transaction platform services for the consumers and the drug sellers in a neutral identity, represented by JD Pharmacy. JD Pharmacy is a B2C online drug market of a famous Chinese third-party B2C platform-JD.COM. Proprietary B2C online pharmacies are mainly electronic transactions between pharmaceutical offline chain enterprises and consumers through proprietary official websites, represented by J1.COM. J1.COM was founded by HuaYuan offline chain Pharmacy in Huarun Group. This article used review data of OTC drug consumers obtained from JD Pharmacy and J1.COM. First, the indicators of consumer satisfaction posted on the B2C e-commerce websites are summarized according to the literature review. At the same time, LDA, an unsupervised machine learning algorithm based on the topic model, is used to discover the factors addressed in the consumers’ reviews. Second, based on a review of the literature, an index of the factors that influence B2C e-commerce website consumers’ satisfaction is used to classify the review factors, and four factor categories of B2C online pharmacy drug reviews are presented. The factor distributions of reviews posted on the websites of the two online pharmacies are compared and analyzed. Third, through analysis using a sentiment dictionary, this article identifies the emotional tendencies of consumers regarding various consumers’ satisfaction factors and compares the emotional tendencies of the consumers in each factor classification. Finally, the conclusions of the article are presented, and the results of the factor discovery, factor classification and sentiment analysis are used to propose rational suggestions for the health services of the two types of B2C online pharmacies. The methodological framework of this article is shown in Fig. 1.

### Consumers’ online satisfaction factors

Szymanski defines a review as the consumer’s perception of his or her entire online shopping experience [35]. The process of creating a consumer review is actually the process in which the consumer explicitly expresses his or her degree of satisfaction with the website. Therefore, identifying the factors that influence consumer satisfaction provides a means of classification of the factors addressed in consumers’ reviews posted on B2C websites, as shown in Table 1 below.

**Table 1:** Factors influencing consumer satisfaction with B2C websites (from the literature)

Author influence factors	Product factors	Staff factors	Logistics factors	Price factors	Information factors	System factors
Lee [36]		√		√		√
Chun-Chun Lin [37]	√	√	√	√	√	√
Xia Liu [38]	√	√	√		√	√
Yooncheong Cho [39]		√				
Gholamreza Torkzadeh [40]	√	√	√	√		√
Ziqi Liao [41]				√		
Szymanski [35]	√					√
Mckinney [42]					√	√
Kim [43]		√	√		√	√
Wolfinbargerhe [44]		√	√			
Timo Koivumaki [45]				√		

Through a review of the relevant literature on the factors affecting consumer satisfaction with B2C e-commerce websites, six factors that affect consumer satisfaction with B2C e-commerce websites were identified. The influencing factors and their definitions are presented in Table 2 below.

**Table 2:** Definitions of influencing factors

Influencing factors	Definition
Product factors	Stable product quality; Reliable product brand
Staff factors	Service attitude and service quality of sales, customer service, and logistics staff
Logistics factors	Dispatch speed; Transport speed; Logistics security; Logistics cost
Price factors	Perceived prices; Competitive prices; Promotions
Information factors	Comprehensive product information; Whether or not product matches product description
System factors	Usability of website; Sound payment mechanism; Payment security

### Data collection

In this article, B2C online pharmacies from which large numbers of consumers purchase drugs and that receive a large number of standardized reviews are divided into two categories: third-party platform-based B2C online pharmacies and proprietary B2C online pharmacies. In this article, we selected two representative online pharmacies in China, JD Pharmacy and J1.COM, and used their websites to obtain OTC drug reviews posted from January 1, 2015 to December 31, 2018 as a corpus.

In this article, a total of 136,630 user reviews was obtained using web crawlers; 72,231 of the reviews were obtained from JD Pharmacy, and 64,399 reviews were obtained from J1.COM.

**Fig. 2:**
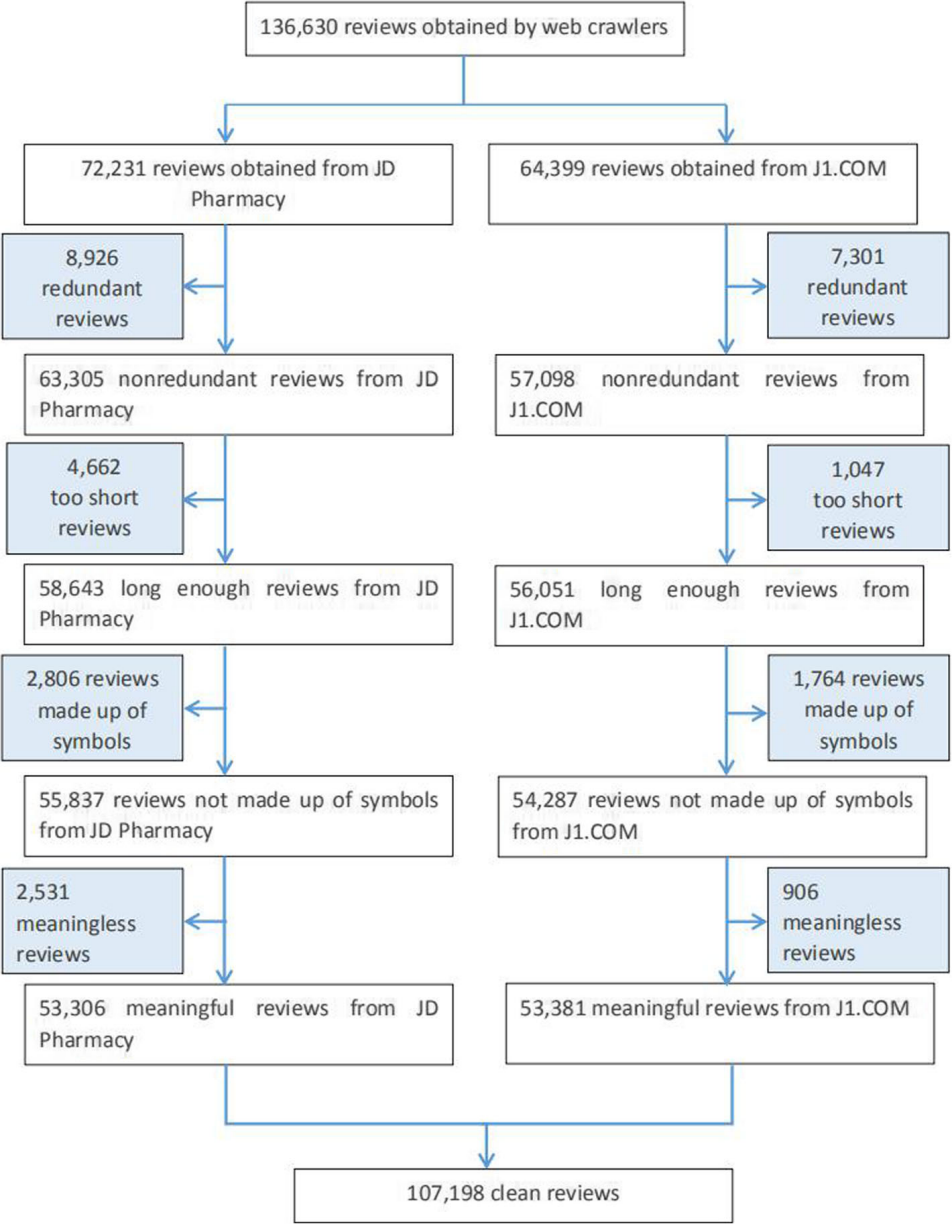
CONSORT-like diagram of data cleaning

The data are cleaned (duplicate, too short, symbols, and meaningless reviews) to reduce the interference of the noisy review data on the LDA factor discovery results. Finally, 107,198 pieces of clean reviews were obtained; 53,306 of these were obtained from JD Pharmacy, and 53,831 were obtained from J1.COM.The CONSORT-like diagram (Fig. 2) shows the data cleaning process.

### Data-driven analysis

#### Data preprocessing

In this article, three steps of preprocessing work were performed on the collected reviews. The first step is that a useful Python kit called Jieba was adopted to segment the Chinese sentences into separate terms [46]. The second step in preprocessing is the deletion of stopwords whose meaning cannot be recognized from the word segmentation. The third step in preprocessing is the merging of synonyms and phrases such as “express” and “logistics” [47].

When the above three steps of data preprocessing had been completed, 19,127 terms remained, and 23% of the terms had been deleted.

#### Factor discovery methods

This article used the LDA (latent Dirichlet allocation) model to classify the factors (topics) of reviews collected from JD Pharmacy and J1.COM. LDA is a Bayesian probability model consisting of a three-layered structure of terms, factors, and document collections [48, 49]. The LDA model considers that the document collection is a mixture of multiple factors and factor is a polynomial distribution within the fixed terms.

The TF-IDF (term frequency-inverse document frequency) model is first used to calculate the weight and the term frequency of each term in the document and to convert each review into a vector. Next, the Gibbs sampling algorithm is used to estimate the posterior of the LDA model parameters [50–52].

#### Sentiment analysis methods

We adopted SnowNLP to carry out sentiment analysis of reviews. SnowNLP is a python kit that specializes in sentiment analysis of Chinese texts. The algorithm of SnowNLP is actually a Naive Bayes algorithm: a simple probabilistic model often used for binary classification of positive texts and negative texts. First, we need to train our data to fit the model. We select 1000 positive and negative reviews each manually. When selecting positive or negative reviews, we use the labels of positive or negative reviews chosen by consumers as reference. Then, we used the selected 2000 reviews to train the model,and then the trained model was used to perform a sentiment analysis on the rest of reviews.

For better understanding the sentiment analysis results, we converted the sentiment scores range from [0,1] to [− 1,1]. If the score is above 0, the emotion of review is regarded as positive; otherwise, it is regarded as negative. The greater the absolute value of the sentiment score of review, the stronger the emotion of review.

## Results

### Factor discovery results

**Fig. 3:**
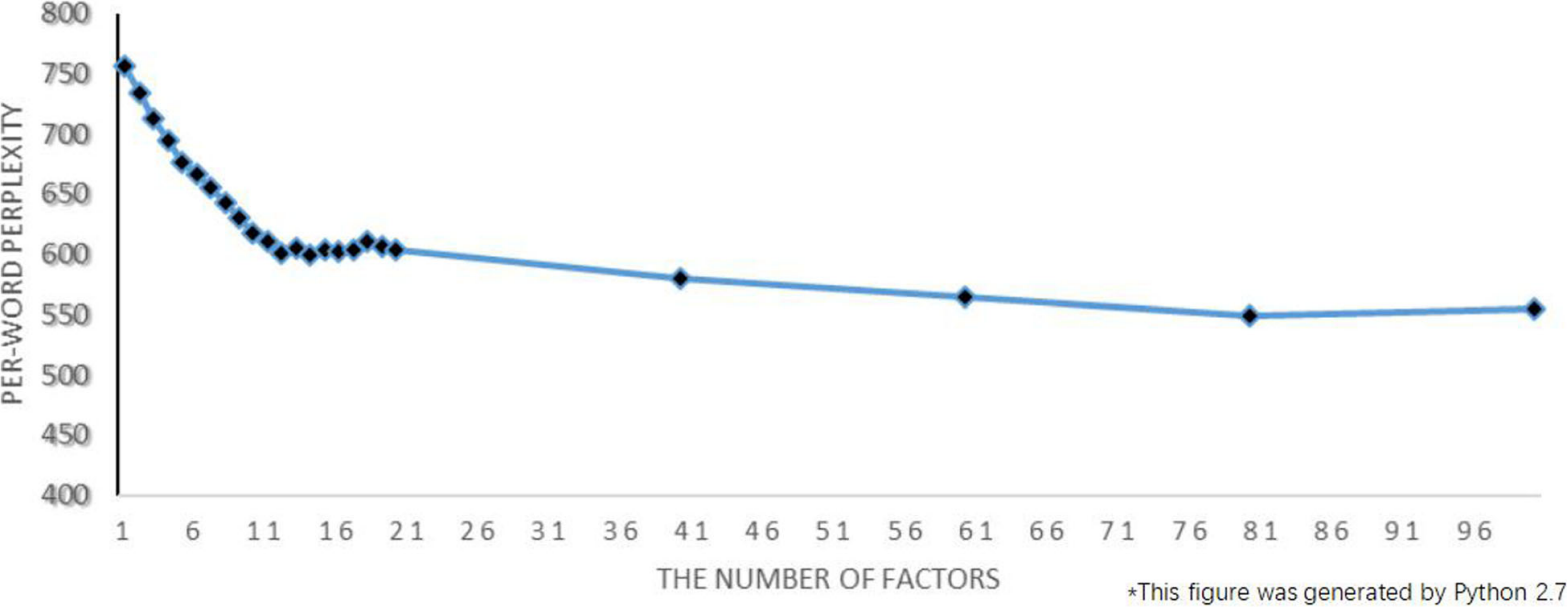
Per-word perplexity as a function of number of factors

Blei, the originator of the LDA model, pointed out that the number of factors in the corpus is determined by its perplexity [48]. The perplexity is the predicted average number of equally likely terms in certain positions. A lower perplexity means a better predictive performance. Figure 3. shows the predictive power of LDA model in terms of the per-term perplexity as a function of number of factors. Perplexity decreases with the increase of factors, and finally tends to be stable. When number of factors is less than 20, the perplexity reaches the minimum at 12. Perplexity decreases much more slowly when number of factors > 20 and it is very difficult to interpret the meaning of factor when the factor number is too large. Therefore, in this article, we set the number of factors to 12 in order to keep a balance between the perplexity and the interpretability. The first 12 keywords in each of the 12 classified factors are selected for the interpretation of that factor. The drug review factor discovery results are shown in Table 3.

**Table 3:** B2C online pharmacy review factors discovered by LDA

Factors	Keywords	Interpretation
Factor 1	logistics, packing, professional, attentively, dry glue, drug name, pharmaceutical factory, arrival of goods, paste, protect, standard, send it over	Professional Logistics Packing (PLP)
Factor 2	genuine, brand, no problem, trust, have faith in, next time, purchase, quality of drugs, guarantee, needs, drug, verify	Trustworthy Drug Quality (TDQ)
Factor 3	inside, box, packing, intact, no damage, logistics, awesome, nice, buy medicine, liquid, bubble wrap, protect	Complete Packing in Logistics (CPL)
Factor 4	expensive, price, more than, Pharmacy, physical store, elsewhere, dosage, spend, offline, hospital, extra money, profit	Expensive (E)
Factor 5	favorable, price, drug, website, save, registration fee, chronic, bring benefit to, cheap, Pharmacy, many times, bottom price	Affordable (A)
Factor 6	dispatch, too slow, speed, too long, wait, unable, receive, drug, bad, for several days, recover, inquire	Slow Dispatch Speed (SDS)
Factor 7	customer service, pass the buck, service, manner, busy, disappointed, solve, problem, adjudicate, irrelevance, unacceptably, cannot understand	Customer service Did Not Solve The Problem (DSP)
Factor 8	slow, logistics, transport, wait, not satisfied with, unable, today, receive, delay, time, for several days, discover	Slow Transport Speed (STS)
Factor 9	fast, speed, logistics, shopping, experience, awesome, pleased, platform, satisfied, receive, today, good	Satisfactory Logistics Speed (SLS)
Factor 10	discount, drug, promotion, satisfactory, high performance-price ratio, awesome, cheap, price, gifts, favorable, bottom price, benefit	Satisfactory Promotion (SP)
Factor 11	take effect, awesome, genuine, confirm, much better, symptom, alleviate, well, Pharmacy, same, dose, satisfied	Satisfactory Drug Effects (SDE)
Factor 12	customer service, quickly, answer, awesome, place an order, response, serious, in time, drug, consumer, at once, inquire	Quick Response of Customer service (QR)

### Factor classification

#### Factor classification results

Based on the review of the factors affecting consumer satisfaction with B2C e-commerce websites, this article analyzes the 12 factors discussed in the previous section and finds that the 12 factors are mainly discussed from four perspectives –logistics, product, price and staff. The factors identified in the review data do not include factors related to an information and system perspective. The reviews of the pharmaceutical e-commerce websites represented by JD Pharmacy and J1.COM include little discussion of information or system factors. It may be that e-commerce has operated in a mature mechanism and that the e-commerce websites chosen for analysis are readily accessible and easy to use. The use of the websites, the integrity and authenticity of the product information, payment security and information security have reached a certain standard and are relatively mature and stable; because consumers are quite accustomed to this, there is little discussion of these factors.

**Fig. 4:**
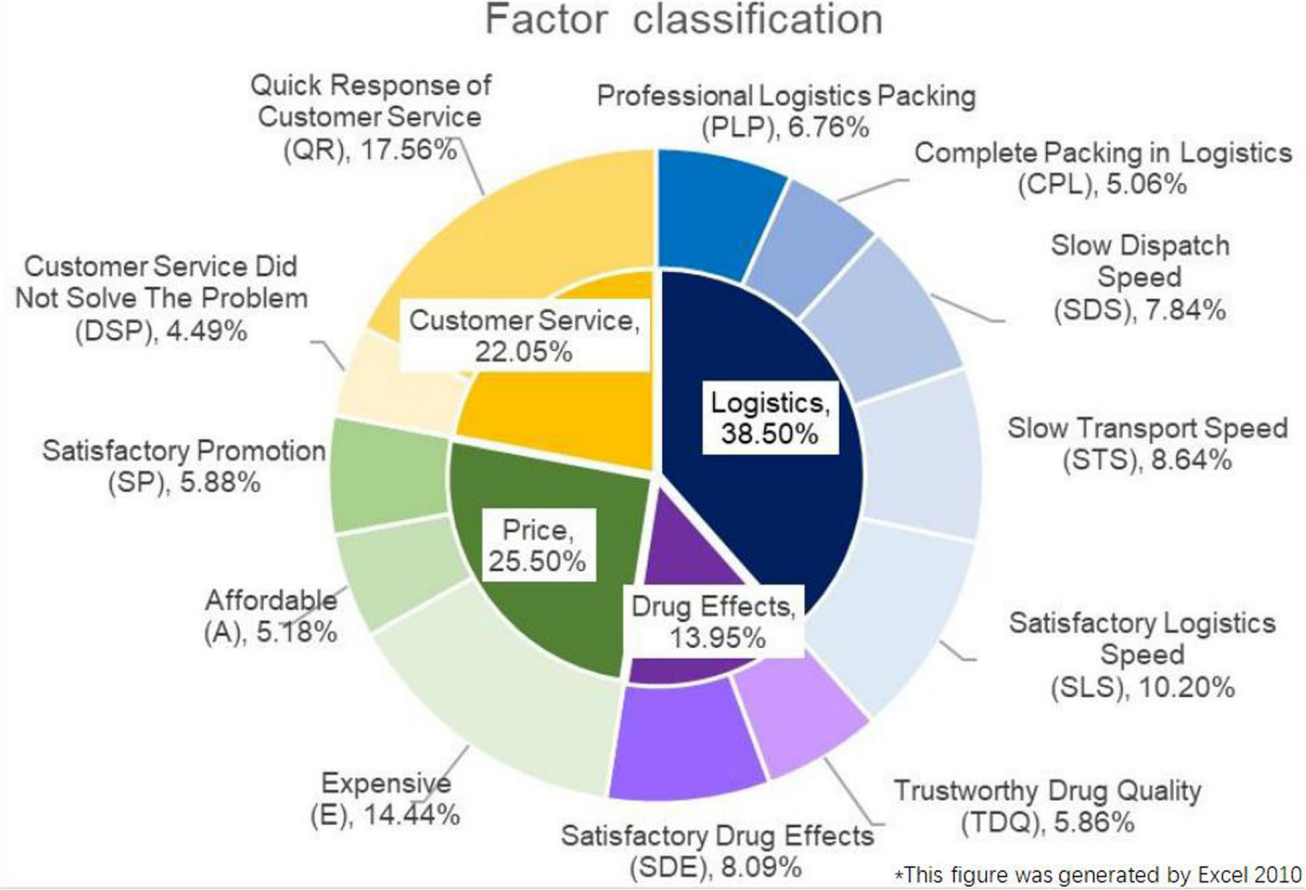
Percentage of reviews in each of the 12 factor categories

As shown in Fig. 4 above, among the 12 factors, QR, E, and SLS accounted for the greatest proportion of the reviews, and the majority of reviews on JD Pharmacy and J1.COM dealt with one or more of these three factors. QR represents the factor Quick Response of Customer service, E represents the factor Expensive, and SLS represents the factor Satisfactory Logistics Speed.

The proportions of drug reviews that fall into the categories of logistics, drug effects, drug price, and customer service are shown in Fig. 4. The figure shows that 5 of the factors are related to logistics; these factors also yield the most relevant drug reviews and account for 38.5% of the total reviews. The number of factors related to drug prices is second highest, with 3 factors, and reviews related to drug prices make up 25.5% of the total reviews. Customer service and drug effects each have 2 related factors; reviews related to drug effects account for a smaller percentage (13.95%) of the total reviews.

#### Differences in the factor distributions of reviews posted at JD pharmacy and J1.COM

**Fig. 5:**
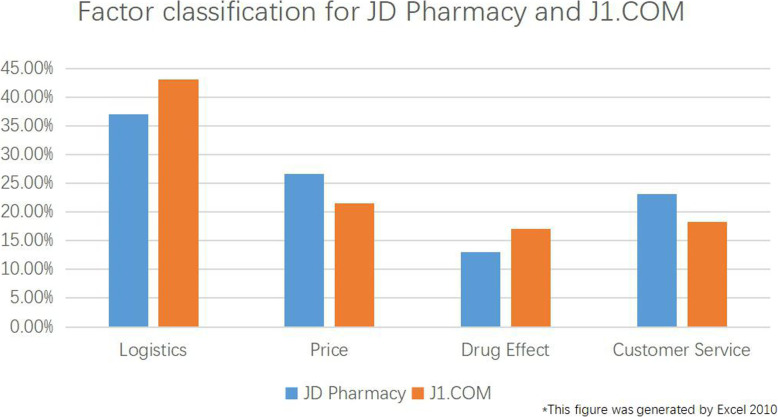
Factor classification for JD Pharmacy and J1.COM

**Fig. 6:**
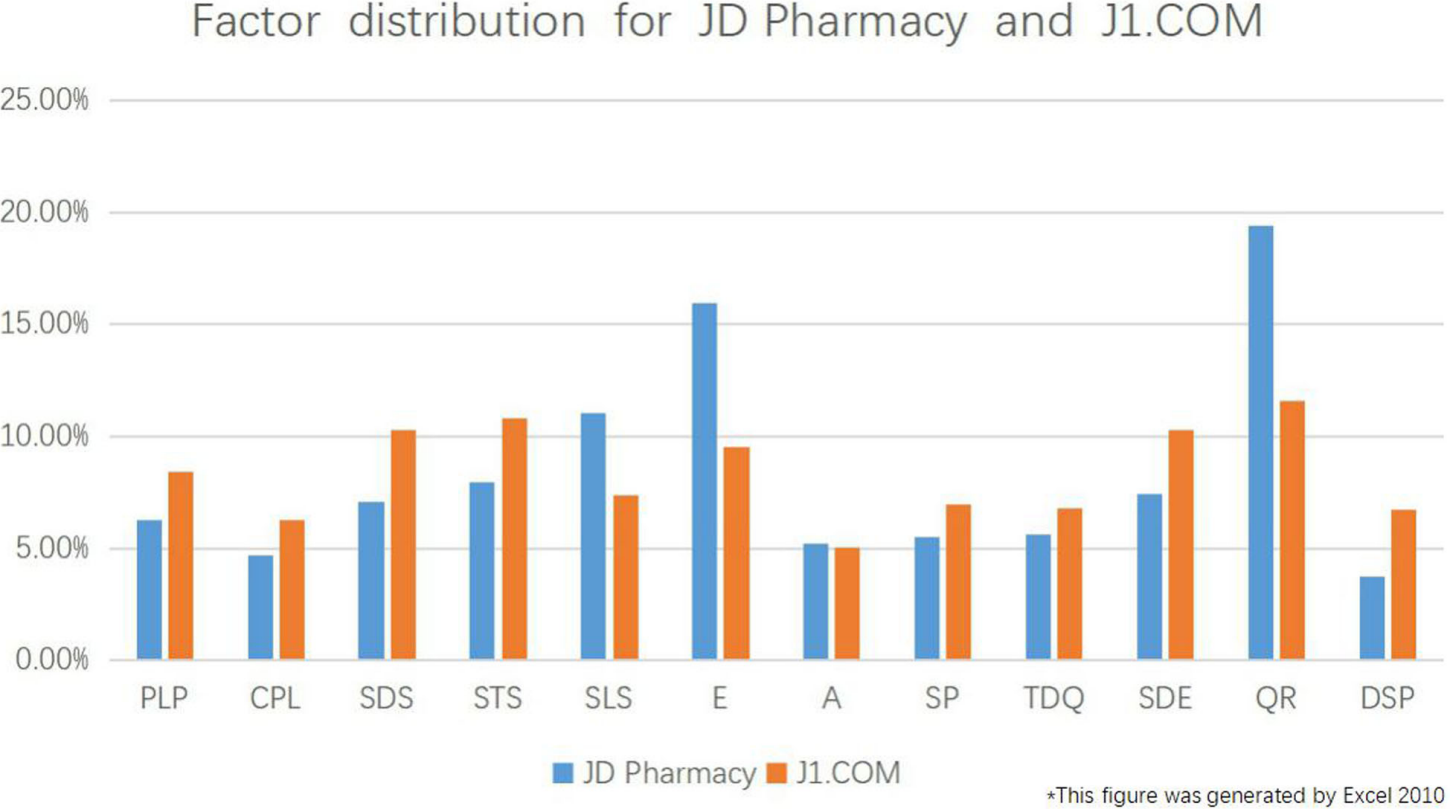
Factor distribution for JD Pharmacy and J1.COM

A comprehensive analysis of Figs. 4, 5 and 6 shows the following:

1. When purchasing medicines, consumers pay the most attention to logistics, followed by drug prices and customer service, and they pay the least attention to drug effects.
2. The proportion of reviews dealing with the factor of logistics is higher at J1.COM than at JD Pharmacy, mainly because consumers engage in extensive discussion of the slow dispatch and transport provided by J1.COM.
3. With respect to the evaluation of drug prices, the number of reviews dealing with the factor of drug prices is much greater at JD Pharmacy than at J1.COM, and there are fewer reviews on the Satisfactory Promotion factor at JD Pharmacy than at J1.COM.
4. With respect to the evaluation of customer service, the proportion of reviews dealing with the factor Quick Response of Customer service is much larger at JD Pharmacy than at J1.COM, and the proportion of reviews with the factor Customer Service Did Not Solve the Problem is smaller at JD Pharmacy than at J1.COM.

### Sentiment analysis

#### Sentiment analysis results

The final results in Table 4 shows that consumers are really satisfied with the two B2C online pharmacies, as the positive sentiment proportion is approximately 90.71%.

**Table 4:** The results of the sentiment analysis of the online pharmacy reviews

Sentiment Polarity	Pharmacy	Count	Percentage	Total
Negative Sentiment	JD Pharmacy	5139	9.64%	9.29%
	J1.COM	4818	8.95%	
Positive Sentiment	JD Pharmacy	48,167	90.36%	90.71%
	J1.COM	49,013	91.05%	

**Fig. 7:**
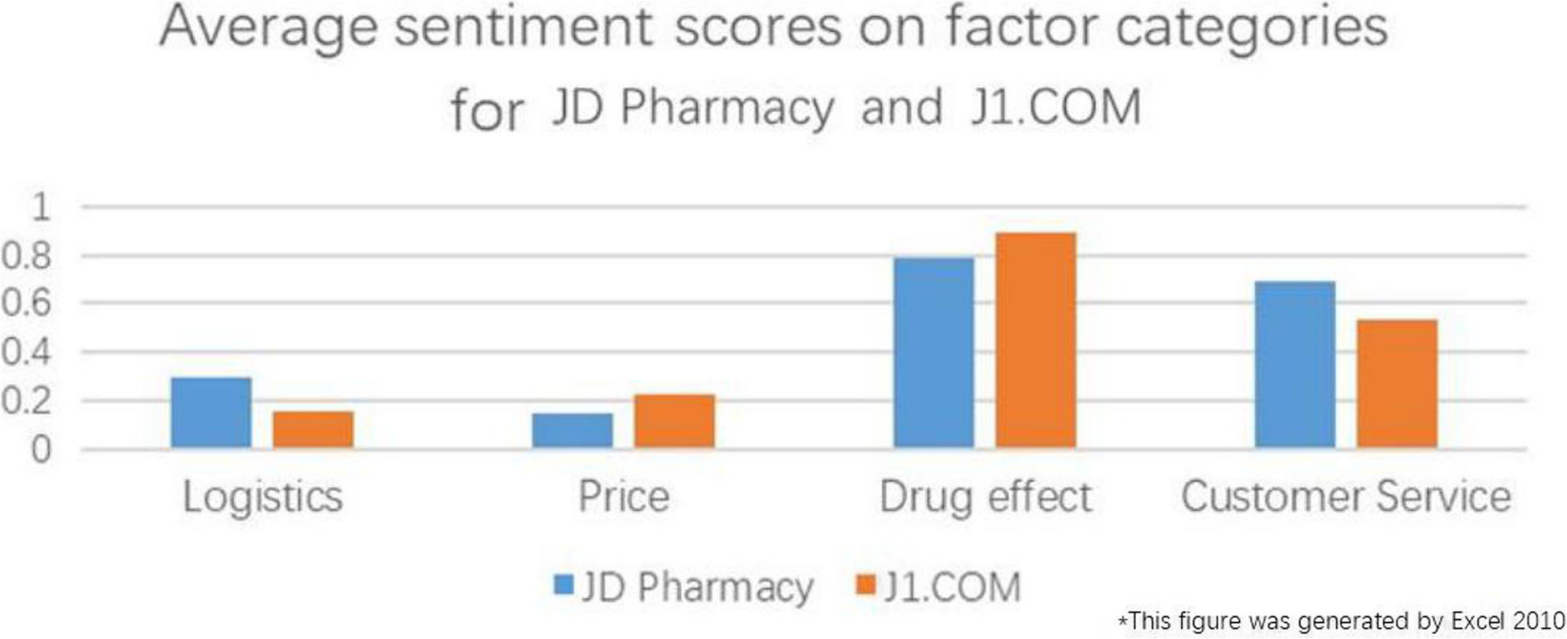
Average sentiment scores on factor categories for JD Pharmacy and J1.COM

**Fig. 8:**
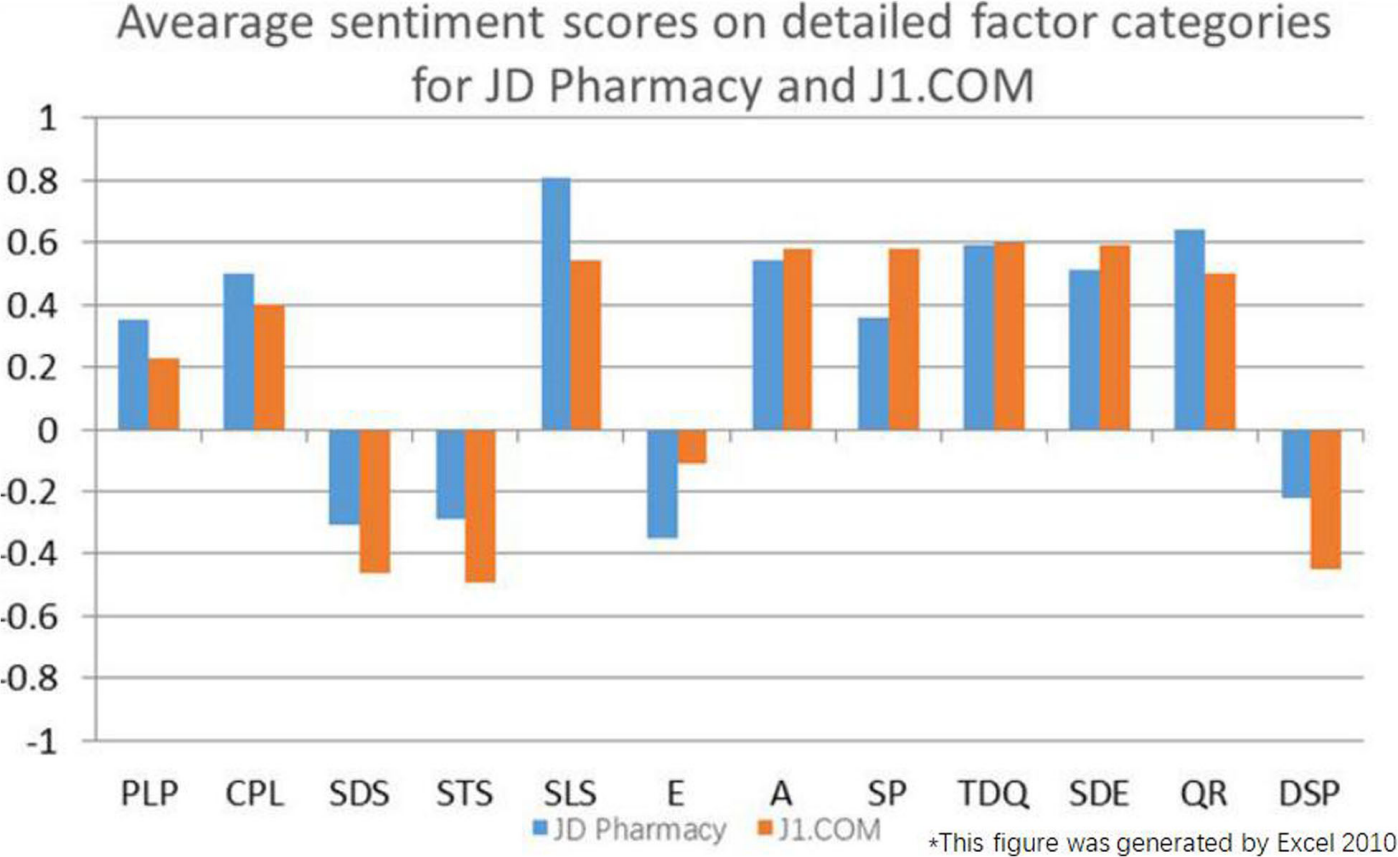
Average sentiment scores on detailed factor categories for JD Pharmacy and J1.COM

A comprehensive analysis of Table 4, Figs. 7 and 8 shows the following:

1. Consumers still maintain positive sentiment for JD Pharmacy and J1.COM. The consumers are satisfied with the drug effects and with the customer service provided by JD Pharmacy and J1.COM. However, there are still some opinions on logistics and drug prices.
2. The logistics and customer service provided by JD Pharmacy are more satisfying to consumers than those provided by J1.COM. The drug prices and drug effects obtained through J1.COM are more satisfying to consumers than those obtained through JD Pharmacy.
3. Positive sentiment for JD Pharmacy regarding logistics speed and customer service response is far greater than that for J1.COM, but JD Pharmacy’s negative sentiment on drug prices is higher than that of J1.COM. The positive sentiment for J1.COM regarding the Satisfactory Promotion factor is greater than that for JD Pharmacy.

#### Evaluation of sentiment analysis results

**Fig. 9:**
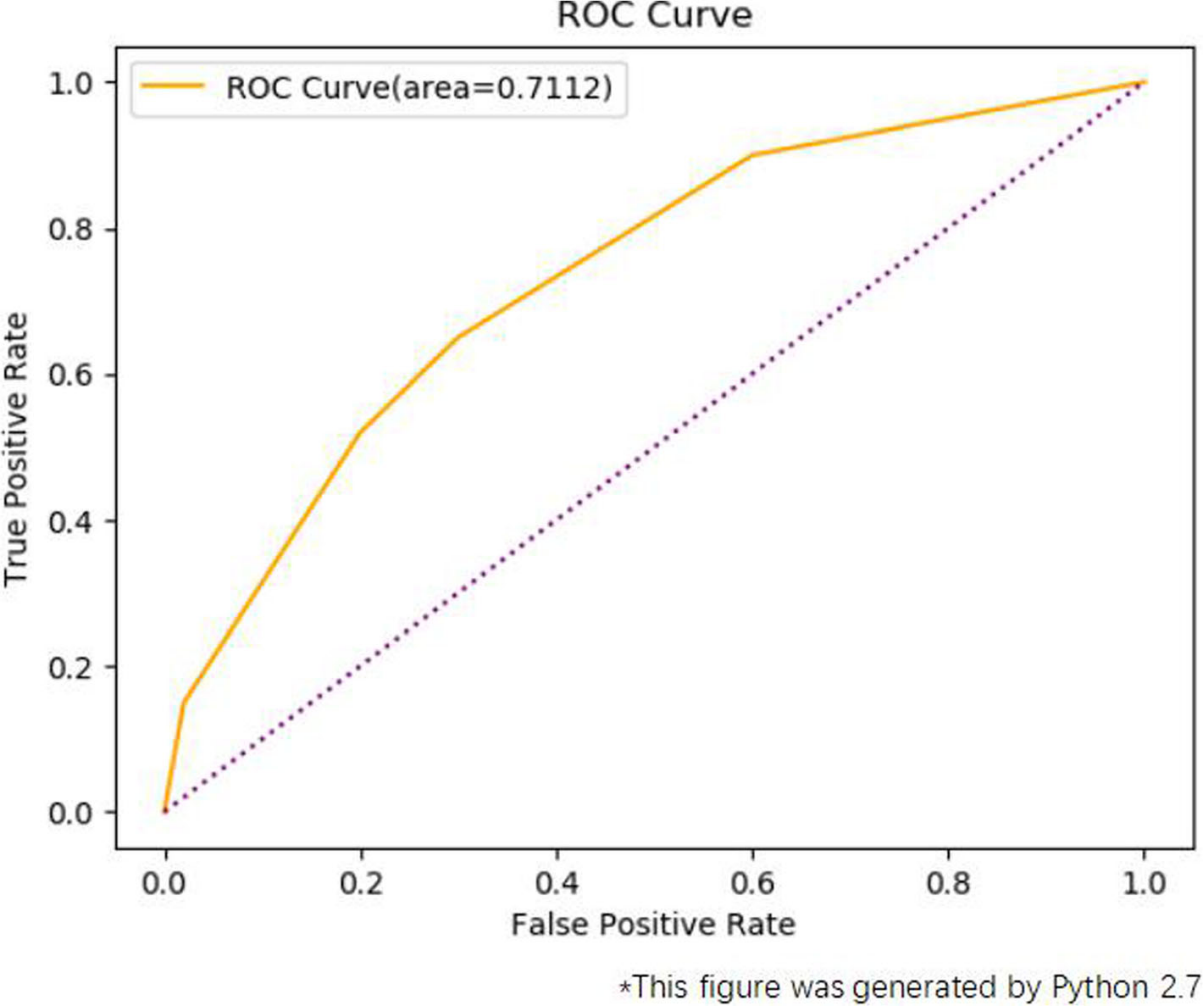
ROC curve for evaluating the sentiment analysis

To evaluate the accuracy of our model performance, we employed the receiver operating characteristic curve (ROC) to obtain the true positive rate and the false positive rate. The true positive rate means that the rate of positive comments which are correctly identified as positive by the algorithm. While the false positive rate means that the rate of negative comments which are mistakenly identified as positive. Firstly, we randomly selected 500 reviews labeled as positive or negative by two researchers and we used these labeled data as the test set. Then, we used the sentiment scores from SnowNLP as the prediction set. After preparing the test set and the prediction set, the ROC curve could be obtained and Area Under Curve (AUC) could be calculated. AUC represents the accuracy of the classifier. If the value of the AUC is between 0.5 and 1, the accuracy of this classifier is better than that of a random guess. In our case, the AUC is 0.7112, which indicates that the result of the sentiment score is satisfactory. Figure 9. shows the ROC curve of our article.

## Discussion

In this article, an algorithm based on the use of the LDA topic model to obtain the factors of B2C online pharmacy reviews was proposed. The 12 factors of B2C online pharmacies were mined and classified into four major factors – logistics, drug prices, drug effects, and customer service. The results of data mining show that consumers pay the most attention to logistics when purchasing drugs, followed by drug prices and customer service, and that they pay the least attention to drug effects.

In reviews on J1.COM, consumers extensively discuss the slow dispatch and transport speed. The logistics of proprietary B2C online pharmacies are a problem that needs special attention. Although proprietary B2C online pharmacies are professional in terms of medicine and professional packing experience, they must rely on third-party logistics because they do not have their own delivery services. This makes it difficult to control the delivery time and the logistics speed. For many years, JD Pharmacy has been proud of its self-built logistical system, which uses multiple warehouses and direct distribution, so the speed of its logistics can often satisfy consumers.

Concerning the reviews of drug prices, J1.COM is a proprietary B2C online pharmacy formed by an offline pharmacy and offers a greater price advantage than JD Pharmacy. As a major feature of e-commerce, low-cost and varied promotional activities are also of particular concern to consumers. Consumers often compare the prices of drugs on e-commerce websites with the prices at offline pharmacies, and online pharmacies usually offer a price advantage.

The reviews of customer service reflect the fact that the diversified integrated sales of home appliances, 3C and other products; its customer service staff is also more adequate and offers better customer service response speed and service quality compared with that of proprietary e-commerce B2C online pharmacies. Due to the lifting of the ban on online pharmacy in China for not so long, consumers may have questions about the quality of drugs and the mechanisms of purchase. Because they need timely responses from customer service, consumers pay more attention to customer service.

In the reviews of drug effects, consumers basically produce positive evaluations for both JD Pharmacy and J1.COM. On one hand, because JD Pharmacy and J1.COM have been well known in China for many years and regardless of whether they are third-party platform-based B2C online pharmacies or proprietary online pharmacies, they are approved and supervised by the government. They offer genuine guarantees. Since proprietary B2C online pharmacies are often professional medical websites, their ability to recommend appropriate drugs based on symptoms is more professional than that of third-party platform-based B2C online pharmacies, so consumers will be more satisfied.

This article has many practical theoretical and managerial implications. First, this article comprehensively uses machine learning methods and theoretical analysis to explore the factor classification and sentiment of B2C online pharmacy consumers’ reviews. For unsupervised factor mining, previous studies mainly used predefined theoretical models and structural equations based on questionnaire data or methods using coded text analysis under unscheduled models. These two methods, which are actually artificial or semimanual predefined coding methods, are time-consuming and laborious, especially when the research includes more than 100,000 pieces of data, and the efficiency of using the manual method is very low. This article uses an unsupervised machine learning algorithm to automatically identify the factors of B2C online pharmacy consumer reviews based on the LDA model. Then, based on a literature review of the factors affecting consumer satisfaction with B2C online pharmacies, the factor discovery results are divided into four major categories.

Second, this article is of great significance with respect to the positioning of consumers’ needs among the two types of B2C online pharmacies, the continuous improvement of the functions of B2C online drug sales, and the improvement of health services level.

The current work indicates that the most important task for both third-party platform-based B2C online pharmacies and proprietary B2C online pharmacies is to enhance the logistics level, improve the delivery and transportation speed, and develop self-built logistics as much as possible. At the same time, online pharmacies can also cooperate with offline pharmacies to realize the Online to Offline (O2O) mode of pharmaceutical e-commerce. Due to the denser characteristics of offline pharmacies, the efficiency of distribution can be improved by means of offline pharmacies [53].

B2C e-commerce has an obvious price advantage because it uses flat transaction channels and has fewer circulation links than do offline stores. B2C online pharmacies should continue to maintain their price advantage. Additionally, aging is showing an increasing trend in China. There are many consumers with chronic diseases, and the demand for pharmaceutical products is high. For some chronic diseases that are treated using drugs with high repurchase rates or drugs that need to be kept at home, the two types of B2C online pharmacies can increase the consumer viscosity or consumer repurchase rate through regular sales.

Customer service should pay attention to cultivating employees’ service attitudes. In particular, proprietary B2C online pharmacies should improve the timeliness of their customer service responses and their problem-solving abilities. Third-party platform-based B2C online pharmacies should especially improve the basic expertise on drugs. If necessary, they should hire professional pharmacists to work in customer service who can answer questions in a professional manner and thereby improve consumer satisfaction, loyalty and trust. For problems involving these aspects, B2C online pharmacies should analyze the causes of consumer concerns and correct their strategies in a timely manner. In the era of big data, a complete customer relationship management (CRM) system should also be established. China has a large population, and the establishment of consumer health records still has great room for development and application in the future [54].

This article shows consumer satisfaction in online pharmacies from a unique and interesting perspective but it also has a number of limitations. First, data in our article were crawled from only two Chinese online pharmacies and the result may be slightly biased. Some consumers also doubt whether the website will retain all the real consumers’ opinions, which means that these online pharmacies will filter out some strong negative comments for commercial purposes. Therefore, we should try our best to expand our data source and improve the quality of data. Second, online pharmacies are still in the initial stage of development in China. However,in some western countries, there are many well-developed online pharmacies like Walgreens and CVS. So we can look further into the services of these advanced pharmacies and carry out more comparisons with growing pharmacies in China.

## Conclusions

Consumers still maintain positive opinions of online pharmacies. However, some opinions on logistics and drug prices are expressed.

The most important task for online pharmacies is to improve logistics. It is better to develop self-built logistics. Both types of online pharmacies can improve consumer viscosity by implementing marketing strategies. With regard to customer service, focusing on improving employees’ service attitudes is necessary.

## Data Availability

The datasets used in the current article are available from the corresponding author on reasonable request.

## Abbreviations

B2C: Business to Customer
LDA: Latent Dirichlet Allocation
OTC: Over-the-counter
WHO: World Health Organization
AHP: Analytic Hierarchy Process
TF: Term frequency
IDF: Inverse document frequency
ROC: Operating characteristic curve
AUC: Area Under Curve
O2O: Online to Offline
CRM: Customer relationship management
